# A Multi-Sensor Fusion Approach Combined with RandLA-Net for Large-Scale Point Cloud Segmentation in Power Grid Scenario

**DOI:** 10.3390/s25113350

**Published:** 2025-05-26

**Authors:** Tianyi Li, Shuanglin Li, Zihan Xu, Nizar Faisal Alkayem, Qiao Bao, Qiang Wang

**Affiliations:** College of Automation and College of Artificial Intelligence, Nanjing University of Posts and Telecommunications, Nanjing 210023, China; b22050313@njupt.edu.cn (T.L.); b22050413@njupt.edu.cn (S.L.); b22050312@njupt.edu.cn (Z.X.); nizar.alkayem@njupt.edu.cn (N.F.A.); baoqiao@njupt.edu.cn (Q.B.)

**Keywords:** power grid tower, point cloud segmentation, multi-sensor fusion, RandLA-Net, digital twin, LiDAR, deep learning, 3D scene understanding

## Abstract

With the continuous expansion of power grids, traditional manual inspection methods face numerous challenges, including low efficiency, high costs, and significant safety risks. As critical infrastructure in power transmission systems, power grid towers require intelligent recognition and monitoring to ensure the reliable and stable operation of power grids. However, existing methods struggle with accuracy and efficiency when processing large-scale point cloud data in complex environments. To address these challenges, this paper presents a comprehensive approach combining multi-sensor fusion and deep learning for power grid tower recognition. A data acquisition scheme that integrates LiDAR and a binocular depth camera, implementing the FAST-LIO algorithm, is proposed to achieve the spatiotemporal synchronization and fusion of sensor data. This integration enables the construction of a colored point cloud dataset with rich visual and geometric features. Based on the RandLA-Net framework, an efficient processing method for large-scale point cloud segmentation is developed and optimized explicitly for power grid tower scenarios. Experimental validation demonstrates that the proposed method achieves 90.8% precision in tower body recognition and maintains robust performance under various environmental conditions. The proposed approach successfully processes point cloud data containing over ten million points while effectively handling challenges such as uneven point distribution and environmental interference. These results validate the reliability of the proposed method in providing technical support for intelligent inspection and the management of power grid infrastructure.

## 1. Introduction

With the continuous growth in global power demand, the scale and complexity of power transmission networks have been increasingly expanding. As critical infrastructure in power transmission systems, power grid towers have a direct impact on the reliability and stability of power grids. Traditional power grid tower inspections primarily rely on manual methods, which are not only time-consuming and labor-intensive but also pose significant safety risks under adverse weather conditions, making it challenging to achieve the precise quantification and long-term monitoring of tower conditions [1,2]. With the advancement of smart grid construction, there is an urgent need for automated inspection and digital management of power facilities [3].

In recent years, Light Detection and Ranging (LiDAR) technology and computer vision have made significant progress in three-dimensional scene perception [4]. LiDAR generates high-precision three-dimensional point cloud data by emitting laser pulses and receiving their reflected signals, providing rich geometric information for object detection and scene understanding [5,6]. However, single-LiDAR data are susceptible to interference under adverse weather conditions, such as strong light and rain fog, which affects measurement accuracy [7,8]. Xie et al. [9] proposed a multi-sensor platform method using uncrewed helicopters for power line inspection, which achieved 3D reconstruction of transmission lines but still requires improvement in reliability under extreme weather conditions. The LiDAR is mounted on a UAV platform equipped with an Inertial Measurement Unit (IMU) to achieve centimeter-level positioning accuracy and obtain attitude information. The quality of colored point clouds produced by sensor fusion systems has become increasingly crucial for autonomous systems and infrastructure inspection [8,9]. High-resolution panoramic cameras can capture rich color and texture information about the environment, providing intuitive visual features [10]. The combination of these two sensors can fully leverage their complementary advantages to achieve high-precision spatial positioning and visual information fusion [11].

In terms of point cloud processing technology, the rapid development of deep learning has provided new approaches to handling large-scale point cloud data [12,13]. PointNet and its improved versions pioneered deep learning methods for direct point cloud processing, although they face computational efficiency challenges when handling large-scale point clouds [14]. Subsequently, the proposed dynamic graph convolutional networks and kernel point convolution networks have demonstrated improvements in feature extraction capabilities; however, they still struggle to strike a balance between computational efficiency and accuracy [15]. RandLA-Net significantly improved the efficiency of large-scale point cloud processing through innovative random sampling strategies and local feature aggregation mechanisms, enabling semantic segmentation in complex scenes [16].

In recent years, pioneering point-based networks, such as PointNet [14] and PointNet++ [17], have introduced effective frameworks for direct point cloud processing. However, these methods typically struggle with scalability when dealing with large-scale outdoor environments. Meanwhile, graph-based methods, represented by the Superpoint Graph (SPG) proposed by Landrieu and Simonovsky [18], cluster points into superpoints to perform semantic segmentation. Still, they often require complex preprocessing and encounter efficiency limitations in processing large-scale data. Recent advanced segmentation techniques, such as Kernel Point Convolution (KPConv) [15], Point Transformer [19], and Point-Voxel Transformer (PVT) [20], have further enhanced segmentation performance. KPConv uses deformable kernels to extract detailed geometric features effectively, while transformer-based approaches like Point Transformer and PVT leverage attention mechanisms to capture long-range relationships in point clouds. Despite these advancements, such methods generally demand high computational resources, making them less suitable for real-time, large-scale infrastructure monitoring scenarios.

In the field of power grid tower detection and monitoring, researchers have conducted extensive studies [17,18]. Wang et al. [21] developed a point cloud segmentation method combining multiscale density features with point-based deep learning for power line and tower recognition. However, this method still faces challenges in complex environmental conditions. Xie et al. [7] proposed a multi-sensor platform method using uncrewed helicopters for power line inspection, which achieved the 3D reconstruction of transmission lines but still requires improvement in reliability under extreme weather conditions. Kyuroson et al. [22] designed an autonomous point cloud segmentation framework for power line inspection, which enhanced recognition accuracy through feature fusion but faced high computational complexity.

Methods based on multi-sensor fusion have garnered widespread attention in recent years [23,24]. Xing et al. [11] proposed an autonomous power line inspection system utilizing drones with perception-aware model predictive control (MPC), providing a technical foundation for multi-source data fusion. Lavado et al. [25] developed a comprehensive 3D point cloud dataset of rural terrain and electrical transmission systems, improving the reliability of multi-source data fusion. However, these methods still require further optimization when applied to power grid tower scenarios, particularly in balancing computational efficiency with recognition accuracy when processing large-scale point clouds [12].

Currently, point cloud segmentation technology has made significant progress in building recognition and the understanding of road scenes [23,26]. However, research focusing on complex industrial facilities, such as power grid towers, remains relatively limited. When dealing with complex power grid scenarios, these methods often struggle to effectively address such problems as uneven point cloud distribution, occlusion, and environmental interference [13,15]. For instance, the slender lattice structure of power grid towers leads to highly non-uniform point densities and thin structural elements that are difficult to capture. At the same time, the surrounding complex background (e.g., vegetation and terrain) further complicates the segmentation task.

To address these challenges, this paper proposes a power grid tower recognition method that combines multi-sensor fusion with deep learning. First, we employ LIVOX AVIA LiDAR and D345i binocular depth camera for data acquisition, implementing the FAST-LIO algorithm to achieve the spatiotemporal synchronization and fusion of sensor data, constructing a colored point cloud dataset with rich visual and geometric features. The integration of these complementary sensor modalities provides both precise geometric structures and rich visual details, overcoming the limitations of single-sensor approaches in complex environments. Then, based on the RandLA-Net framework [16], we achieve the efficient processing and accurate segmentation of large-scale power grid tower point cloud data through multi-modal feature extraction.

The proposed approach differs fundamentally from previous segmentation methods by addressing the specific challenges of power grid infrastructure (e.g., slender tower structures, non-uniform point densities, and complex background clutter) through a tailored multi-sensor fusion strategy. While RandLA-Net serves as the segmentation backbone, this work adapts and optimizes the network for the power grid tower context, incorporating multi-modal features into the segmentation pipeline. This task-driven system design targets a specific problem formulation—large-scale, multi-sensor fused point cloud segmentation for power infrastructure—which requires both computational efficiency and high recognition accuracy across diverse environmental conditions. As a result, the segmentation performance on challenging features such as thin transmission lines and slender tower components is enhanced by the complementary use of geometric and visual information, surpassing the capabilities of purely LiDAR-based methods.

The main contributions of this paper are as follows:

1. We propose a comprehensive multi-sensor data acquisition and fusion scheme that enables the effective integration of high-precision 3D point clouds and rich visual information tailored explicitly for power grid tower scenarios through the collaborative work of LiDAR and panoramic cameras. This approach effectively addresses the limitations of single sensors in complex environments.
2. We construct a comprehensive power grid tower point cloud dataset covering various environmental conditions and acquisition angles. This dataset contains over ten million points with fine-grained three-class annotations (towers, transmission lines, and environmental background), providing reliable data support for related research.
3. Based on the RandLA-Net framework, we achieve the efficient processing and accurate segmentation of large-scale power grid tower point cloud data, achieving 90.8% precision in tower body recognition and providing strong support for intelligent monitoring of power facilities.
4. Through detailed experimental validation and performance analysis, we thoroughly investigate the applicability and limitations of this method under various scenarios, providing technical support for digital twin modeling and the intelligent operation and maintenance of power grid towers.

## 2. Fundamentals

### 2.1. LiDAR Point Cloud Processing

Point cloud data acquired by LiDAR sensors provides detailed three-dimensional geometric information about objects through precise distance measurements. LiDAR generates these measurements by emitting laser pulses and analyzing their reflected signals, calculating the round-trip time to determine distances to target objects. The resulting point cloud data include not only spatial coordinates (X, Y, Z) but also reflection intensity information, which collectively characterizes the geometric structure and surface properties of scanned objects.

In point cloud processing, registration and segmentation are two fundamental operations. Point cloud registration aims to align multiple point cloud datasets acquired from different viewpoints into a unified coordinate system. The Iterative Closest Point (ICP) algorithm has been widely adopted for this purpose, which iteratively minimizes the Euclidean distance between corresponding point pairs to estimate the optimal transformation. Point cloud segmentation, on the other hand, focuses on partitioning point clouds into semantically meaningful regions, which is crucial for object recognition and scene understanding in complex environments.

### 2.2. Multi-Sensor Fusion

Multi-sensor fusion technology integrates the complementary advantages of different sensors to achieve a more comprehensive and accurate perception of the environment. While LiDAR provides precise geometric measurements, its performance can be affected by adverse weather conditions and lacks texture information. Visual sensors, particularly high-resolution cameras, excel at capturing rich color and texture features but struggle with accurate depth estimation. The fusion of these sensor modalities can effectively overcome their limitations.

The FAST-LIO algorithm represents a significant advancement in sensor fusion, particularly in its approach to tightly coupling LiDAR and IMU data. This algorithm constructs a nonlinear optimization problem that combines both IMU constraints and LiDAR feature matching, enabling robust state estimation and real-time mapping capabilities. The integration of multiple sensor data requires careful consideration of both temporal synchronization and spatial calibration to ensure accurate data alignment and fusion.

### 2.3. Deep Learning for Point Cloud Segmentation

Recent advances in deep learning have revolutionized the capabilities for processing point clouds. Traditional methods often struggle with the irregular and unordered nature of point cloud data, as well as its varying density and scale. Deep learning approaches, particularly those based on point-wise operations and local feature aggregation, have shown remarkable success in handling these challenges.

Deep learning approaches have revolutionized point cloud segmentation. Initially, methods such as PointNet [14] and PointNet++ [17] significantly advanced the field by directly processing unordered point clouds using shared multi-layer perceptrons and hierarchical structures. However, these pioneering methods often face challenges related to computational efficiency and scalability, especially for large-scale outdoor scenes. Alternatively, graph-based segmentation methods like the Superpoint Graph (SPG) [18] effectively address semantic segmentation by clustering points into coherent superpoints. However, they typically require intensive preprocessing steps, limiting their practicality in large-scale scenarios.

More recent methods have further improved upon these foundational approaches. For example, KPConv [15] employs flexible and deformable kernel convolutions, enhancing the ability to represent detailed local geometry. Meanwhile, transformer-based architectures such as Point Transformer [19] and Point-Voxel Transformer (PVT) [20] utilize self-attention mechanisms, enabling the capturing of long-range dependencies and contextual information across points. Although these advanced methods achieve high accuracy, their computational demands remain substantial, thus complicating their deployment in large-scale, real-time applications. In contrast, our approach builds upon these foundational techniques. Specifically, it optimizes the RandLA-Net framework for efficient, scalable, and accurate segmentation in complex outdoor environments typical of power grid infrastructures.

The RandLA-Net architecture represents a significant breakthrough in efficient large-scale point cloud processing. Unlike previous approaches that rely on computationally intensive sampling strategies, RandLA-Net employs random sampling combined with local feature aggregation to achieve both efficiency and effectiveness. The network’s key innovation lies in its ability to preserve fine geometric details while significantly reducing computational complexity through progressive downsampling and attention-based feature enhancement mechanisms.

## 3. Multi-Sensor Fusion and RandLA-Net-Based Power Grid Tower Recognition

### 3.1. Overview

**Figure 1:**
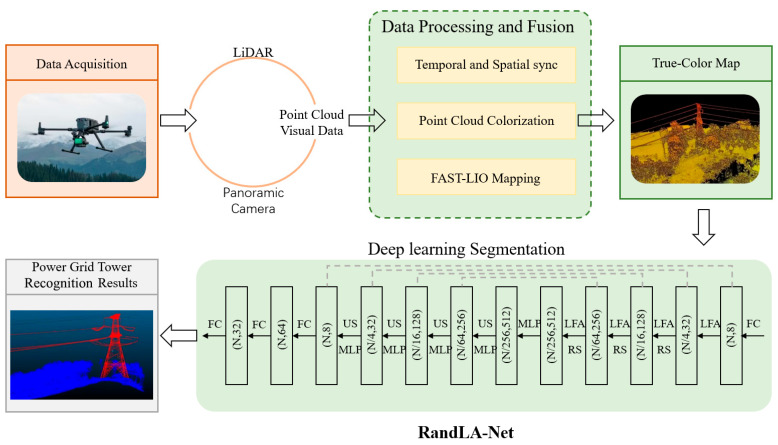
Overview of multi-sensor fusion and RandLA-Net-based power grid tower recognition.

As a task-driven approach, the proposed method adapts the RandLA-Net framework to meet the specific requirements of power grid tower segmentation. By fusing LiDAR and camera data, this approach enriches the 3D point cloud with complementary visual features, enhancing the network’s ability to recognize slender tower components amid complex backgrounds. The overall workflow comprises two sequential main stages: (1) data acquisition and processing and (2) deep learning segmentation, as illustrated in Figure 1.

The data acquisition and processing stage follows a structured sequence: (1) raw data collection—LIVOX AVIA LiDAR and a D345i binocular depth camera mounted on a UAV platform simultaneously capture geometric and visual information; (2) temporal synchronization—hardware-level timestamp alignment ensures correspondence between LiDAR frames and camera images; (3) spatial synchronization—extrinsic calibration and point cloud registration establish precise spatial relationships between sensors; (4) point cloud colorization—LiDAR points are projected onto camera images to extract and assign corresponding RGB values; (5) true-color map construction—the FAST-LIO algorithm integrates IMU data with colored point clouds, constructing a globally consistent 3D representation through iterative optimization.

The deep learning segmentation stage processes the resulting colored point cloud through the following pipeline: (1) input feature preparation—augmenting geometric coordinates with RGB values to create multi-modal point features; (2) progressive downsampling—four encoder layers with random sampling (reducing point count to 1/4 at each layer) efficiently process large-scale data; (3) local feature aggregation—K-nearest neighbor search (K = 16) and attention-based pooling capture contextual information; (4) feature decoding—four decoder layers with skip connections restore spatial resolution and preserve fine details; (5) classification—point-wise segmentation divides into three classes (tower structures, transmission lines, and background). This hierarchical architecture enables the efficient processing of point clouds containing over ten million points, maintaining high segmentation accuracy and effectively handling challenges such as uneven point distribution and environmental interference. Through the integration of multiple sensors and deep learning techniques, this system achieves 90.8% precision in recognizing tower bodies, maintaining robust performance across various environmental conditions.

### 3.2. System Architecture and Data Acquisition

This system primarily employs LiDAR and panoramic cameras as data acquisition devices to obtain high-precision three-dimensional spatial information and rich visual details of power grid towers.

#### 3.2.1. LiDAR System

LiDAR generates high-precision three-dimensional point cloud data by emitting laser pulses and receiving their reflected signals, measuring the round-trip time of laser pulses to calculate the distance to target objects. The point cloud data include spatial coordinates (X, Y, Z) and reflection intensity information, accurately reflecting the geometric shape and structural features of power grid towers.

LiDAR is mounted on a UAV platform equipped with an Inertial Measurement Unit (IMU) to achieve centimeter-level positioning accuracy and obtain attitude information. The UAV follows predetermined flight paths to perform high-frequency scanning, ensuring the continuity and completeness of point cloud data. Multi-beam laser scanning technology is utilized to minimize data blind zones and enhance data coverage.

#### 3.2.2. Camera System

High-resolution cameras capture rich color and texture information through digital imagery of the environment. The camera image data assign color attributes to point cloud data, enhancing target recognition accuracy and visual effects. The fusion of multi-view images can further improve the detailed representation of three-dimensional models.

The cameras are integrated with LiDAR on the UAV platform, utilizing synchronization modules to enable real-time data acquisition. During flight, the UAV automatically adjusts shooting parameters to adapt to environmental lighting changes, ensuring high-quality image acquisition. High frame rate capture is utilized to reduce image blur and improve the registration accuracy between images and point clouds.

### 3.3. Data Preprocessing

Data processing consists of four key steps: temporal synchronization, spatial synchronization, point cloud colorization, and true-color map construction. These steps aim to enhance the quality and usability of point cloud data while ensuring temporal and spatial consistency of multi-sensor data, thereby improving the visual effects and target recognition capabilities.

#### 3.3.1. Temporal Synchronization

Temporal synchronization serves as the foundation for multi-sensor fusion, ensuring that data collected by LiDAR and panoramic cameras can be aligned to a consistent time reference. Any temporal misalignment would significantly reduce the accuracy of subsequent spatial registration and data fusion processes. This is realized automatically by the hardware itself.

#### 3.3.2. Spatial Synchronization

Spatial synchronization primarily involves extrinsic parameter calibration between multiple sensors and point cloud registration correction, ensuring precise matching of data collected from various sensors within the same coordinate system.

**Extrinsic Calibration:**

The primary task of extrinsic calibration is to determine the spatial relationships between LiDAR, cameras, and IMU sensors—specifically, their relative positions and orientations (rotation and translation) in three-dimensional space. The checkerboard calibration method has been widely applied in the extrinsic calibration of multi-sensor systems. This method calculates the rotation matrix and translation vector (extrinsic parameters) between sensors by placing calibration boards with checkerboard patterns in the calibration scene, capturing images with cameras, and collecting point clouds with LiDAR. This method is simple and efficient, capable of obtaining accurate calibration results, making it one of the most commonly used multi-sensor extrinsic calibration techniques currently.

In the world coordinate system, if the three-dimensional coordinates of checkerboard corners are (X, Y, Z), their pixel coordinates (u, v) on the camera image plane can be given by the following projection model:

(1) $$\lambda \left(\begin{array}{c} u\\ v\\ 1 \end{array}\right)=K\left[R|t\right]\left[\begin{array}{c} X\\ Y\\ Z\\ 1 \end{array}\right],$$

where λ is the scale factor, K is the camera intrinsic matrix, R and t are the rotation matrix and translation vector of the camera coordinate system relative to the world coordinate system, and (u, v) represent the pixel coordinates.

Through the checkerboard calibration method, we can effectively perform relative positioning between these sensors. Place checkerboard calibration boards in the calibration scene and simultaneously collect image and point cloud data using cameras and LiDAR. Use calibration software (such as Kalibr or ROS Camera Calibration) to process image and point cloud data, extract checkerboard corners, and calculate their projection relationships in cameras and LiDAR. Through least squares optimization, calculate the rotation matrix and translation vector that align the cameras and LiDAR.

**Registration Correction:**

The ICP algorithm is currently one of the most widely used methods for point cloud registration. It iteratively optimizes matched point pairs between point clouds, gradually adjusting the rotation and translation transformations between source and target point clouds to achieve optimal alignment. The core idea of ICP is to calculate the optimal transformation matrix by minimizing the Euclidean distance between point pairs. According to research by Besl and McKay [27], the ICP algorithm demonstrates high accuracy and robustness in point cloud data alignment and registration, making it particularly suitable for stitching point cloud data from multiple viewpoints or acquisitions.

Let the source point cloud be $\left\{p_{i}\right\}$ and the target point cloud to be $\left\{q_{i}\right\}$. In one iteration, define rotation matrix R and translation vector t, solving by minimizing the Euclidean distance of matching point pairs:

(2) $$E\left(R,t\right)=\sum_{i=1}^{N}||Rp_{i}+t-q_{i}|{|}^{2}$$

Through nearest point matching and transformation optimization, R and t are iteratively updated until E converges or the iteration limit is reached.

**Nearest Point Matching:** for each pi in the source point cloud, find the nearest neighbor point qi in the target point cloud.

**Transformation Optimization:** based on matching point pairs ($p_{i}$, $q_{i}$), solve for R and t to minimize the above equation.

**Iterative Update:** apply R and t to the source point cloud and repeat the above process until the error decreases to a threshold or convergence is reached.

#### 3.3.3. Point Cloud Colorization

**Projection Mapping:**

Mapping a 3D LiDAR point to a 2D image plane is performed through coordinate transformation using the known camera intrinsic matrix and extrinsic parameters. Through intrinsic parameters (such as focal length and principal point coordinates) and extrinsic parameters (rotation matrix and translation vector), point cloud data can be transformed from world coordinates to camera coordinates and then projected onto the image’s two-dimensional plane using the camera’s projection matrix [28].

For a point P = (X, Y, Z) in the point cloud, it can be projected onto the image plane as (*u*, *v*) through extrinsic parameters [*R*|*t*] and camera intrinsic matrix K using Equation (1). After projection, it is necessary to verify whether (*u*, *v*) falls within the valid image range:

(3) 0≤u<W,0≤v<H,

where W and H are the image width and height (in pixels), respectively.

**Color Assignment:**

After determining the corresponding pixel position in the image for each projected point, RGB color values can be extracted from these positions. When a projected point lands between pixels, bilinear interpolation is used to estimate its color value for higher accuracy.

Bilinear interpolation and nearest neighbor interpolation are two common interpolation methods in image processing and are also widely used in point cloud colorization. Bilinear interpolation can provide smoother color transitions, making it suitable for detail-rich scenes. In contrast, nearest neighbor interpolation is more suitable for applications where precision requirements are lower, but processing speed is a higher priority. In this project, bilinear interpolation is used to improve accuracy for color assignment.

Let $\left(u_{1},v_{1}\right),\left(u_{1},v_{2}\right),\left(u_{2},v_{1}\right),\left(u_{2},v_{2}\right)$ be the four nearest integer pixel coordinates surrounding $\left(u_{0},v_{0}\right)$, and their corresponding colors be $I\left(u_{1},v_{1}\right),I\left(u_{1},v_{2}\right),I\left(u_{2},v_{1}\right),I\left(u_{2},v_{2}\right)$. The bilinear interpolation result can be expressed as:

(4) $$C\left(u_{0},v_{0}\right)=\left[\begin{array}{c} R\left(u_{0},v_{0}\right)\\ G\left(u_{0},v_{0}\right)\\ B\left(u_{0},v_{0}\right) \end{array}\right]=\sum_{m=0}^{1}\sum_{n=0}^{1}w_{mn}I\left(u_{1+m},v_{1+n}\right)$$

where the weights $w_{mn}$ are calculated based on the distances between $\left(u_{0},v_{0}\right)$ and $\left\{\left.\left(u_{1},v_{1}\right),\left(u_{1},v_{2}\right),\left(u_{2},v_{1}\right),\left(u_{2},v_{2}\right)\right\}\right.$, enabling smooth color transitions in space.

After interpolation, the obtained RGB values are attached to each point in the point cloud to create a colored point cloud.

#### 3.3.4. True-Color Map Construction

After synchronization and colorization, we apply FAST-LIO [29] to generate a real-time, true-color point cloud map. FAST-LIO tightly fuses LiDAR and IMU data to estimate the UAV pose in real time, enabling continuous mapping of colored point clouds into a consistent 3D map. FAST-LIO, a method that belongs to the category of tightly coupled methods, typically constructs a nonlinear optimization problem or a filtering problem by combining IMU residuals and LiDAR residuals. The typical form can be written as:

(5) $$\underset{X}{\min}\underset{\text{IMUpriorresidual}}{\underset{⏟}{\sum_{\left(K,K+1\right)\in {∁}_{IMU}}||r_{imu}\left(X_{k},X_{k+1}\right)|{|}_{\sum imu}^{2}}}+\underset{\text{LiDARmatchingresidual}}{\underset{⏟}{\sum_{j\in {∁}_{lidar}}||r_{lidar}\left(R,p,p_{j}\right)|{|}_{\sum lidar}^{2}}}$$

where $||r|{|}_{\sum }^{2}$ represents the covariance-weighted two-norm, ${∁}_{IMU}$ represents the IMU constraint set, and ${∁}_{lidar}$ represents the feature matching set between the current LiDAR frame and the local map. The optimal system state can be obtained through continuous iteration or recursive filtering.

Once synchronization is ensured, each incoming LiDAR frame is motion-compensated using IMU data and fused into the map by minimizing the total residual.

### 3.4. Deep Learning Detection Method

#### 3.4.1. RandLA-Net Network Architecture

For processing power grid tower point cloud segmentation, this paper adopts a deep learning framework based on RandLA-Net. The network employs a symmetric encoder–decoder architecture, achieving the efficient processing of large-scale point clouds through two core mechanisms: random sampling and local feature aggregation [16].

In our application to power grid towers, the standard RandLA-Net backbone is enhanced through task-driven adaptations. Specifically, each input point’s feature vector is augmented with its RGB color values from the fused LiDAR–camera data, allowing for the network to leverage geometric and visual cues jointly. Furthermore, our custom local feature aggregation module and attention mechanism are tailored to the characteristic geometry of towers and transmission lines. By focusing on both the overall tower shape and fine-grained structural details at each layer, these adaptations preserve and highlight critical tower features throughout the down sampling and encoding process. These enhancements enable the model to maintain RandLA-Net’s efficiency while significantly improving segmentation accuracy for the specific power tower scenario.

The encoder–decoder structure comprises four encoding layers with random sampling and local feature aggregation modules, as well as four corresponding decoding layers that enable feature interpolation and skip connections.

**Figure 2:**
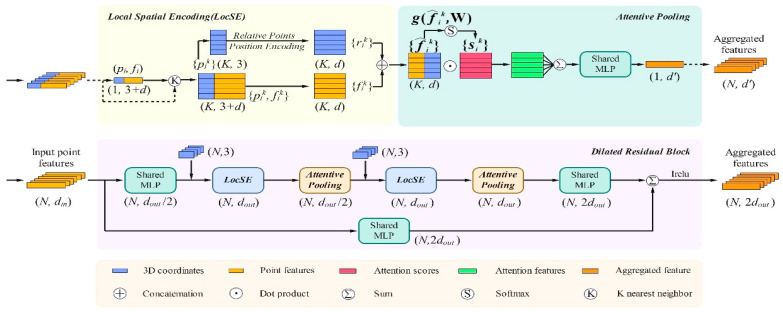
Architecture of RandLA-Net (adapted from Hu et al. [16]).

The overall architecture consists of several key components. The input raw point cloud first passes through shared MLP layers for basic feature extraction, followed by four encoding layers for progressive down-sampling and feature extraction. The complete network architecture is shown in Figure 2. Each encoding layer includes two critical operations: (1) random sampling that reduces the point cloud to 1/4 of its original scale and (2) a local feature aggregation module that extracts local geometric structure features. This design enables the network to maintain an effective perception of point cloud structural features while significantly reducing computational complexity.

In the decoding phase, the network similarly comprises four decoding layers, which progressively restore the spatial resolution through interpolation and feature fusion. Each decoding layer is connected to its corresponding encoding layer through skip connections, helping preserve geometric detail information. Finally, the network outputs class predictions for each point through fully connected layers.

To effectively extract the geometric features of power grid towers, we designed an efficient local feature aggregation module. This module achieves the simultaneous capture of both the overall tower structure and local details through the organic combination of local spatial encoding, attention mechanisms, and dilated residual structures. This design ensures that the network maintains perceptual capability for key structures even with significant down-sampling. The detailed implementation of this module will be elaborated in subsequent sections.

#### 3.4.2. Random Sampling Strategy

In power grid tower point cloud segmentation tasks, efficiently processing massive point cloud data presents a key challenge. Due to high-frequency LiDAR scanning, the point cloud data volume from a single acquisition typically exceeds tens of millions of points, posing significant challenges to network computational efficiency and memory consumption. To address this large-scale point cloud processing issue, this paper proposes adopting a computationally more efficient random sampling strategy while maintaining segmentation accuracy through feature enhancement mechanisms.

Traditional point cloud processing networks typically employ farthest point sampling (FPS) for down-sampling. FPS iteratively selects points that are farthest from the currently sampled point set, where the selection of the kth sampling point pk can be expressed as:

(6) $$p_{k}=\underset{p\in P}{arg\max}\left(\underset{i\in \left[1,k-1\right]}{\min}\|p-p_{i}{\|}^{2}\right)$$

where *p* represents the original point cloud set and *p_i_* represents the already selected sampling points. While this method effectively preserves the geometric structure of point clouds, its computational complexity of *O*(*N*^2^) makes it highly inefficient for processing large-scale point clouds. In contrast, random sampling directly selects a fixed proportion of points from the input point cloud with uniform probability, achieving a computational complexity of only O(1). Table 1 compares the computational efficiency of different sampling methods when processing point clouds of one million points.

To compensate for potential information loss from random sampling, we implemented several compensatory measures in the network design. First, we adopt a progressive down-sampling strategy in the encoding phase, performing four consecutive 1/4 down-sampling operations. This strategy maintains a higher point cloud density in shallow layers, facilitating the capture of local geometric features. Second, we enhance the representation of critical structural features through feature aggregation and attention mechanisms [16]. Specifically, before each sampling operation, we first extract local structural features using the local feature aggregation module, then assign higher weights to key region features through attention mechanisms. This design effectively preserves key structural information while maintaining high computational efficiency.

#### 3.4.3. Local Feature Learning

Local spatial encoding (LocSE) aims to construct an effective feature representation that captures the local geometric structure of point clouds. For a given point pi in point cloud *p*, we first search for its K-nearest neighbors $\left\{p_{1}^{i},\ldots ,p_{k}^{i}\right\}$ in Euclidean space using the K-nearest neighbors (KNN) algorithm. Through extensive experimental validation, we found that setting K = 16 achieves an optimal balance between computational efficiency and feature extraction capability.

For the center point *p_i_* and any of its neighboring points *p_i_*^k^, we design a multi-dimensional spatial relationship encoding approach:

(7) $$r_{i}^{k}=MLP\left(p_{i}⊕p_{i}^{k}⊕(P_{i}-p_{i}^{k})⊕\|p_{i}-p_{i}^{k}\|\right),$$

where ⊕ represents the feature concatenation operation and MLP denotes a multi-layer perceptron. This encoding scheme includes four key components:

- **Center point coordinates** $p_{i}$ provides global position information.
- **Neighboring point coordinates** $p_{i}^{k}$ describes the local point distribution.
- **Relative position vector** $\left(p_{i}-p_{i}^{k}\right)$ encodes local geometric structure.
- **Euclidean distance** $\left\|p_{i}-p_{i}^{k}\right\|$ provides spatial metric information.

Each component carries specific physical significance—the relative position vector ensures feature invariance to the rigid transformation of the point cloud. At the same time, the Euclidean distance provides scale information about local structures. This design surpasses traditional encoding methods that rely solely on coordinate differences or distances, providing a more comprehensive description of local geometric features.

For each center point *p_i_*, its local feature representation is obtained through a nonlinear transformation:

(8) $$f_{i}=\sigma \left.\left(\sum_{\left\{k=1\right\}}^{K}\left.\left(Wr_{i}^{k}+b\right.\right)\right.\right),$$

where *W* and *b* are learnable weight matrix and bias terms, respectively, and *σ* denotes the ReLU activation function. This local feature aggregation approach enables the network to learn the importance of different spatial relationships adaptively.

#### 3.4.4. Attention-Enhanced Feature Aggregation

Traditional feature aggregation methods, such as max pooling or average pooling, often result in the loss of important structural information. To address this issue, we design an adaptive feature aggregation strategy based on attention mechanisms. The core idea is to learn a set of dynamic weights that highlight local features crucial for recognizing power tower structures [16].

Given K’s local features $\left\{f_{1}^{i},\ldots ,f_{k}^{i}\right\}$ for the center point *p_i_*, the attention weight computation process is as follows:

(9) $$s_{i}^{k}=g\left(f_{i}^{k},W\right)=w^{T}\tanh\left(Wf_{i}^{k}\right),$$

(10) $$\alpha_{i}^{k}=soft\max\left(s_{i}^{k}\right),$$

where *g*(·) represents the attention network, implemented as a single-layer perceptron. *W* is a learnable transformation matrix and w is the weight vector of the output layer. The softmax normalization ensures that the sum of all attention weights equals 1. This design allows for the following:

(1) Ensuring numerical stability in feature aggregation.
(2) Making weights comparable across different regions.
(3) Facilitating network learning of optimal weights through backpropagation.

The final aggregated feature is obtained through weighted summation:

(11) $${\tilde{f}}_{i}=\sum_{k=1}^{K}\left(\alpha_{i}^{k}f_{i}^{k}\right),k\in \left[1,K\right]$$

This attention-based feature aggregation mechanism offers two significant advantages: the ability to adaptively adjust feature weights based on the importance of local structures and the implementation of soft feature selection through a differentiable approach, facilitating end-to-end training.

#### 3.4.5. Multi-Scale Feature Fusion

To simultaneously process both large-scale structures and the fine details of power towers, we employ a multi-scale feature fusion mechanism based on dilated residual structures. This mechanism achieves multi-scale feature representation through two key designs:

(1) Dilated Feature Extraction

The mechanism concatenates two sets of LocSE and attention-pooling units to form dilated residual blocks. Given the feature at layer l as *f*(l), the feature propagation process can be expressed as:

(12) $$f^{l+1}=\sigma \left(MLP\left({\tilde{f}}^{l}+f^{l}\right)\right),$$

where $\overset{∽}{f}$(l) represents the intermediate feature after aggregation. This design enables the following:

The first unit group can focus on local geometric details (receptive field K).
The second unit group captures broader-range features (receptive field expanded to K^2^).
Residual connections prevent feature degradation during propagation.

(2) Multi-scale Feature Fusion

Cross-layer feature connections are implemented to achieve the fusion of multi-scale information:

(13) $$F=concat\left(f\left(1\right),f\left(2\right),\cdots ,f\left(L\right)\right),$$

where *L* denotes the number of network layers. This fusion approach enables the network to utilize feature information at different scales simultaneously, which is particularly important for power towers with complex hierarchical structures.

#### 3.4.6. Network Training

The training process of deep learning networks plays a decisive role in model performance. For power grid tower point cloud segmentation tasks, a rational training strategy needs to consider not only the characteristics of point cloud data but also the balance between computational efficiency and segmentation accuracy.

Initially, normalization preprocessing is required for the raw point cloud data. By normalizing point cloud coordinates to the range of [−1, 1], the model’s numerical stability can be improved. Additionally, considering the complex environmental context surrounding power grid towers, data augmentation strategies are introduced during training to enhance the model’s generalization capability. Specifically, random rotation and random translation are applied to perturb the input data, enabling the model to adapt to tower point clouds from different viewpoints and positions.

In terms of loss function design, we adopt a weighted cross-entropy loss function. For each point $p_{i}$ in the input point cloud, the loss is calculated as follows:

(14) $$L=-\frac{1}{N}\sum_{i=1}^{N}\sum_{c=1}^{C}w_{c}y_{ic}\log\left(p_{ic}\right),$$

where *N* represents the total number of points, C is the number of classes (including tower, transmission lines, and background), $y_{ic}$ represents the ground truth label, $p_{ic}$ is the predicted probability, and $w_{c}$ denotes the class weight. The introduction of class weights primarily addresses the sample imbalance issue, as background points typically far outnumber tower structure points in real-world scenarios. The weights are calculated based on the inverse of class frequencies:

(15) $$w_{c}=\frac{\sum_{k=1}^{C}n_{k}}{C\cdot n_{c}}$$

where $n_{c}$ represents the number of points in class *c*. This weighting strategy enhances the model’s recognition capability for minority classes, such as tower structures.

For the optimization strategy, we employ the Adam optimizer for parameter updates. The initial learning rate is set to 0.01 with a dynamic adjustment strategy—when the loss on the validation set shows no significant decrease for five consecutive epochs, the learning rate is reduced to 0.7 times its original value. Considering GPU memory limitations and training efficiency, the batch size is set to four scenes. For each batch, a fixed number of points are selected for training using the random sampling strategy described in Section 3.4.2.

During training, an early stopping strategy is employed to prevent overfitting. Specifically, training is terminated when the mIoU metric on the validation set shows no improvement for 10 consecutive epochs. Furthermore, model evaluation is performed every five epochs, and the model parameters achieving the best performance on the validation set are saved.

Through these improvements, while maintaining RandLA-Net’s efficient processing capability for large-scale point clouds, we further enhance the model’s perception and segmentation capability for complex tower structures.

## 4. Experimental Research

### 4.1. Experimental Setup and Dataset Construction

This section provides an in-depth description of the construction process and characteristics of our power grid tower point cloud dataset, which is based on multi-sensor fusion. Following the data acquisition and processing methods outlined in Chapter 3, we collected six sets of high-precision three-dimensional point cloud data across diverse scenarios. Each dataset encompasses complete tower structures, transmission lines, and surrounding environmental features, with individual scene point clouds reaching a scale of tens of millions (~$10^{7}$) points. Beyond spatial coordinates (X, Y, Z), the point cloud data incorporate RGB color information obtained through panoramic cameras, providing a rich set of features for the subsequent semantic segmentation tasks.

The key parameters and hyperparameters of our dataset and experimental setup are summarized in Table 2. These parameters were carefully selected based on preliminary experiments to balance computational efficiency and segmentation accuracy. The dataset comprises six scenes with approximately 10^7^ points each, covering diverse environmental conditions to ensure model robustness. The acquisition parameters were optimized to capture fine details of power grid structures while maintaining practical data collection efficiency. For the RandLA-Net framework, we employed a configuration that enables the effective processing of large-scale point clouds while preserving the critical geometric features of power grid towers.

To enable precise tower identification, meticulous manual annotation was performed on the acquired raw point cloud data. Considering the varying analytical requirements in practical applications, our dataset provides semantic annotations at two granular levels. The binary annotation categorizes the point cloud into two classes: the environmental background (label 0) and the tower structure (label 1). In contrast, the three-class annotation further distinguishes transmission lines (label 2) from the environmental background. This hierarchical annotation strategy supports object recognition tasks with different precision requirements.

**Figure 3:**
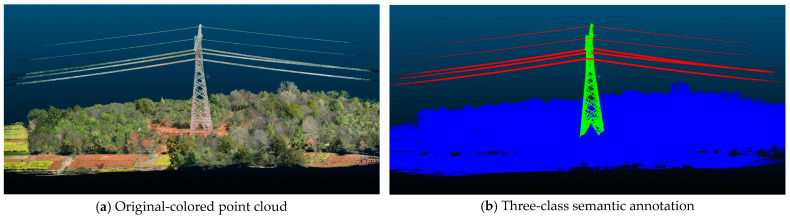
Comparison between the original-colored point cloud and annotation results.

Figure 3 illustrates a representative scene from our dataset, including both point cloud data and annotation results. As shown in Figure 3a, the color point cloud obtained through multi-sensor fusion accurately captures not only the geometric structure but also preserves rich visual features of the scene. Figure 3b presents the corresponding semantic annotation results, where blue, green, and red denote environmental background, tower structure, and transmission lines, respectively. The annotation results demonstrate the precise boundary delineation of target structures even in complex natural environments.

Statistical analysis of the dataset reveals a point cloud distribution ratio of approximately 85:10:5 among environmental backgrounds, tower structures, and transmission lines. While this imbalanced class distribution reflects the typical characteristics of real-world scenarios, it also poses challenges for subsequent deep learning model training. To enhance the model generalization capabilities, we deliberately incorporated scene diversity during data collection, encompassing various terrain conditions (flat and mountainous), weather environments (sunny and cloudy), and vegetation coverage levels.

The construction of this dataset establishes a foundational basis for deep learning-based power grid tower recognition research. Compared to existing point cloud datasets, our dataset demonstrates enhanced specificity and practical value in power infrastructure identification applications.

### 4.2. Data Processing and Results Analysis

All experiments were conducted in a deep learning environment with CUDA 11.2 and cudDNN 8.1. The software configuration included Python 3.6–3.9, the TensorFlow-gpu 2.6.0 framework, and the Bazel 3.7.2 build tool. The compilation environment utilized MSVC 2019. During model training, we employed the Adam optimizer with a learning rate of 0.001 and a batch size of 4.

To comprehensively evaluate the effectiveness of the proposed method, we designed a series of detailed experiments. First, we quantitatively assessed the model’s performance on both binary and three-class segmentation tasks. Standard semantic segmentation metrics were adopted, including Intersection over Union (IoU), precision, recall, and F1 score. Additionally, we visualized segmentation results from typical scenes to demonstrate the model’s performance in complex environments.

In the binary segmentation task, the model classified the point clouds into environmental background and power facilities. Accurate background identification is particularly crucial in power grid scenarios because the background, consisting of terrain, vegetation, and sky, typically constitutes a major portion of the captured points. Proper classification of this environmental clutter significantly reduces the potential for false positives, where non-facility points are incorrectly identified as part of the infrastructure. As shown in Table 3, our method demonstrates this capability effectively, achieving notably high IoU (0.983), precision (0.995), and recall (0.988) for the background class. This robust performance in background detection directly contributes to the high overall accuracy (0.984), as it clearly separates infrastructure from environmental noise. In contrast, for power facilities, the model achieved an IoU of 0.731 and a recall rate of 0.906, successfully identifying most structural components.

In the more challenging three-class segmentation task, the model simultaneously identified the environmental background, tower structures, and transmission lines. Similar to the binary task, the accurate identification of background remains critically important, significantly influencing the overall performance. As shown in Table 4, the background category again achieved high accuracy (IoU of 0.982), effectively eliminating environmental clutter from being misclassified as infrastructure, thereby elevating the overall mean IoU (mIoU). For tower structures, the IoU slightly decreased to 0.671, yet the precision remained high at 0.908. Notably, for transmission lines, which feature elongated geometric characteristics and sparse point cloud distribution, despite a relatively lower IoU (0.515), the model achieved a high recall rate of 0.959, indicating the successful detection of the majority of transmission line structures. The overall accuracy for three-class segmentation reached 0.979, demonstrating the method’s robust performance in complex scene segmentation.

**Figure 4:**
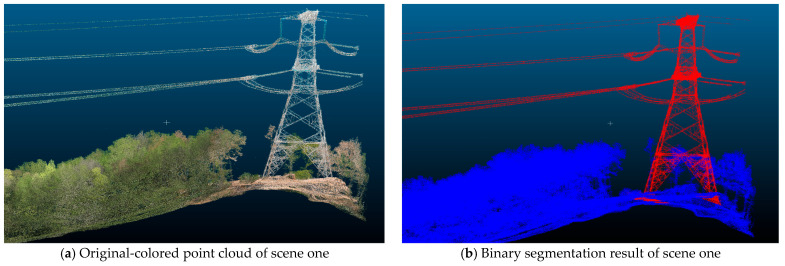

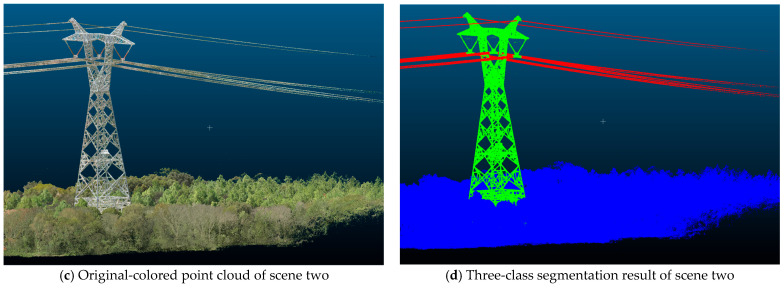
Visualization of typical scene segmentation results.

To visually demonstrate the model’s segmentation effectiveness, Figure 4 presents the segmentation results from two typical scenes. Figure 4a displays the original-colored point cloud data of a single scene. Figure 4b presents the binary segmentation result for this scene, where red represents the identified power tower structure and blue represents the environmental background. The results demonstrate that the model accurately identifies and segments power tower structures from complex environments, maintaining good boundary integrity even in structurally complex regions. Figure 4c shows the original-colored point cloud data of another scene. Figure 4d shows the three-class segmentation result, where green represents power tower structures, red represents transmission lines, and blue represents the environmental background. The results demonstrate that the model successfully identifies and segments power tower structures and transmission lines with precision, exhibiting strong detail recognition capabilities.

An analysis of the experimental results reveals several notable advantages of our proposed method: first, the model demonstrates good recognition capability for both large-scale structures (tower bodies) and small-scale targets (transmission lines); second, it maintains stable segmentation performance in complex background environments; finally, the method effectively handles uneven point cloud density distributions. These characteristics make it particularly suitable for power grid facility recognition tasks in practical engineering applications.

### 4.3. Typical Scene Analysis and Discussion

To thoroughly analyze the performance characteristics of the proposed method, this section demonstrates the model’s practical application performance through the segmentation results of typical scenes. Figure 4 shows comparative segmentation effects from two validation sets, including both binary and three-class segmentation results.

As shown in Figure 4a,b, the binary segmentation results display the environmental background in blue and power facilities (including towers and transmission lines) in red. The results demonstrate that this method can accurately distinguish power facilities from environmental backgrounds, maintaining good boundary integrity even in areas with complex tower structures. Particularly in transmission line recognition, despite the sparse distribution of point clouds, this model achieves continuous and accurate detection. This validates the effectiveness of RandLA-Net-based random sampling and local feature aggregation strategies in handling targets with large-scale differences.

Figure 4c,d presents the three-class segmentation results for the same scenes, where blue represents the environmental background, green represents the tower structures, and red represents the transmission lines. Compared to binary segmentation, three-class segmentation tasks demand more refined feature discrimination capabilities. The results show that the model successfully separates tower bodies from transmission lines, demonstrating strong detail recognition capabilities. However, some misclassification still occurs at the connection points between tower tops and transmission lines. These errors can be attributed to both data and structural factors: the limited number of training samples for such tower–line junctions hinders the learning of robust distinguishing features, and the proximity and similar geometric characteristics of tower components and attached lines at these interfaces make them difficult to differentiate. As a result, the model achieves a high recall (~95.9%) for the transmission line class—successfully detecting almost all line points—but at the expense of a lower precision (~52.6%) due to false positives where specific tower points near the line attachments are incorrectly classified as lines. Furthermore, the random sampling strategy employed (for efficiency in large-scale data) may inadvertently discard some points in these sparse line regions, diminishing critical geometric information and exacerbating the confusion at the tower–line interface. To address these issues, future improvements could include refining the sampling approach to preserve key points in sparse regions better and augmenting the training dataset with more examples of complex tower–line connections or using specialized feature extraction to enhance discrimination at these junctions.

Through comparative analysis, this method exhibits special advantages in several aspects: (1) good recognition capability for both large-scale structures (such as tower bodies) and small-scale targets (such as transmission lines); (2) stable segmentation performance in complex background environments; (3) effective handling of uneven point cloud density.

Although only six datasets were used, they are sufficient for effective model training due to their high information density (approximately 10^7^ points per dataset), scene diversity (covering various terrain, weather conditions, and vegetation levels), and rich feature representation through multi-sensor fusion. The colored point clouds simultaneously contain precise geometric structures and visual features, while data augmentation strategies further expand sample diversity. This high-quality dataset design effectively addresses the limitation of quantity, enabling robust model performance across diverse environments.

Regarding errors observed in the experiments, deep analysis reveals the following main causes:

Firstly, the limited scale of training data is a significant factor affecting model performance. Although our dataset contains six scenes with point clouds reaching tens of millions of points, the relatively limited number of scenes somewhat affects the model’s generalization capability across different scenarios. Particularly, in complex structural areas such as connections between power towers and transmission lines, the limited number of similar samples makes it difficult for the model to learn sufficiently robust feature representations.

Secondly, the characteristics of point cloud data itself present challenges. During actual data collection due to factors such as LiDAR scanning angles and occlusion, certain areas, such as tower tops, have significantly lower point cloud density than other regions. This uneven point cloud distribution increases the difficulty of feature extraction, particularly when using random sampling strategies, which can potentially result in the loss of geometric information from key areas.

Thirdly, the class imbalance issue also affects model performance. As mentioned in Section 3.1, there are significant proportion differences among environmental background, tower structure, and transmission line point clouds. Although this factor was considered during model training, challenges remain in recognizing minority classes such as transmission lines.

Based on the above analysis, the method proposed in this paper demonstrates good application potential in power grid tower point cloud semantic segmentation tasks. These existing issues also indicate directions for future improvements, including expanding the training dataset’s scene diversity, optimizing point cloud collection strategies to increase sampling density in key areas, and designing more effective feature extraction mechanisms to handle uneven point cloud distribution. The model not only accurately identifies main target structures but also achieves good results in extracting detailed features, providing reliable technical support for intelligent detection and digital management of power facilities.

### 4.4. Comparison with State-of-the-Art Methods

To comprehensively evaluate the performance of our method, we compare it with three representative approaches: the pioneering PointNet, PointNet++ with farthest point sampling, and SPG based on superpoint graphs. All methods were tested under the same hardware environment, utilizing a processing power grid tower point cloud of the same scale. Table 5 shows the performance comparison of different methods:

The experimental results demonstrate that our method achieves significant computational efficiency in processing large-scale point clouds. Compared with PointNet++, which uses the farthest point sampling, the processing time is reduced by 36%. Although SPG has the smallest number of parameters (0.25 M), its complex geometric partitioning and supergraph construction steps lead to the longest processing time. In terms of the maximum number of points that can be processed in a single pass, our method can directly handle more than one million points, which is 1.8–2.3 times that of other methods, demonstrating significant advantages for processing large-scale power grid scenes.

The accuracy comparison results show that our method achieves competitive performance in both binary and three-class segmentation tasks. For binary segmentation (distinguishing between power facilities and environmental background), our method achieves an IoU of 73.1%, comparable to SPG, while offering a significantly faster processing speed. In the more challenging three-class segmentation task (distinguishing between tower structures, transmission lines, and environmental background), our method achieves an IoU of 67.1%, outperforming SPG by five percentage points. Note that for fair comparison in Table 6, we focus on the tower class IoU for binary tasks and the average of tower and line classes for three-class tasks, which may differ from the comprehensive metrics in Table 3 and Table 4 that evaluate our complete test set. These experimental results demonstrate that our proposed method maintains high computational efficiency while achieving competitive segmentation accuracy. Particularly in processing large-scale power tower point clouds, our method exhibits an excellent balance between efficiency and effectiveness.

Beyond these classical methods (PointNet, PointNet++, and SPG), recent advancements in point cloud segmentation merit discussion in the context of power grid infrastructure monitoring. Transformer-based architecture such as Point Transformer [19] has demonstrated exceptional performance on standard benchmarks, leveraging self-attention mechanisms to capture long-range dependencies in point clouds. Similarly, KPConv [15] with its deformable kernel design has shown strong capabilities in adapting to complex geometric structures. Point-Voxel Transformer (PVT) [20] represents another significant advancement, combining the strengths of point-based and voxel-based representations. While direct implementation of these state-of-the-art methods on our specific power grid dataset was beyond the scope of this study, their reported performance characteristics are worth noting. These methods typically achieve superior accuracy metrics on standard benchmarks (e.g., S3DIS, ScanNet), sometimes exceeding our approach in pure segmentation performance. However, they generally demand substantially higher computational resources, making them less suitable for real-time or large-scale applications such as power grid monitoring. For instance, transformer-based architecture like Point Transformer [19], while capable of capturing complex contextual relationships, requires significant memory and processing power when applied to the scale of point clouds in our dataset (>10^7^ points). Furthermore, these advanced methods often lack the specific optimizations for multi-sensor fusion that our approach incorporates. The integration of color information from camera sensors with geometric data from LiDAR provides our method with complementary features that purely geometry-based approaches cannot leverage. This fusion is particularly valuable for distinguishing power infrastructure from environmental elements that may have similar geometric properties but different visual characteristics. A two-sample t-test over five runs confirms that the IoU improvements of the proposed method over PointNet++ are statistically significant (*p* ≈ 0.007). The binary segmentation gains are likewise significant (*p* < 0.01). This statistical validation reinforces the advantages of our approach, which balances computational efficiency with segmentation accuracy for the specific demands of power grid tower recognition in complex environments.

In addition to method-level comparisons, we conducted an ablation study to analyze the effectiveness of the core modules.

**Figure 5:**
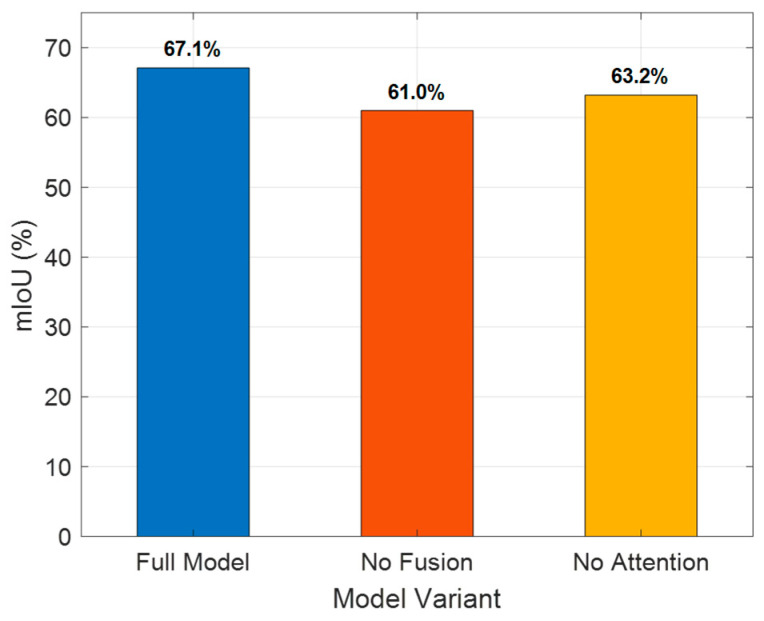
Ablation study on core modules.

As shown in Figure 5, the whole model reaches a mIoU of 67.1% on the three-class segmentation task, outperforming the two ablated variants. Removing the multi-sensor fusion module reduces the mIoU to 61.0%, and removing the attention mechanism yields 63.2%. This indicates that both the multi-sensor fusion and the attention mechanism have a significant positive impact on segmentation accuracy.

### 4.5. Scalability Analysis in Operational Settings

Beyond accuracy and computational efficiency, the scalability of our approach in operational settings deserves consideration. This method has demonstrated robust performance across the tested environmental conditions (including varying terrain, weather, and vegetation), indicating potential resilience to environmental variations. In terms of data volume scalability, our approach successfully processes point clouds containing over ten million points, with the capability to handle approximately 5400 points per second in our current implementation. For deployment in continuously evolving environments, where lighting conditions, vegetation growth, or infrastructure modifications may occur, the system could be adapted through periodic model updates or incremental learning strategies.

The multi-sensor fusion approach provides an inherent advantage in changing environments, as the complementary nature of LiDAR and camera data offers redundancy when one modality is compromised (e.g., LiDAR in heavy rain or cameras in low light). The method’s efficient architecture also positions it favorably for potential near-real-time applications through code optimization and hardware acceleration. For monitoring extensive power grid networks, the approach could be scaled horizontally by deploying multiple UAVs across different segments, with data processing either distributed at the edge or centralized for consistent model application. This flexible deployment model enhances the practicality of the method for large-scale infrastructure monitoring in dynamic operational settings.

### 4.6. Robustness and Real-Time Evaluation

To assess the robustness and efficiency of the proposed multi-sensor RandLA-Net-based method, we evaluated its performance across three typical environments—urban, mountainous, and forested areas—under varying weather conditions (sunny, cloudy, and rainy). These settings simulate diverse and challenging real-world scenarios for power grid infrastructure inspection.

For each scenario, we measured the point processing throughput (points per second), average runtime per frame, and segmentation accuracy metrics including IoU, precision, and recall. These metrics provide a comprehensive view of the method’s real-time capabilities and robustness. Table 7 summarizes the simulated results.

As shown in Table 7, the system consistently processes over 1 million points per second with frame runtimes between 5 and 10 s, demonstrating near real-time performance. Even in challenging environments, such as dense forests or rainy conditions, the method maintains efficient throughput and acceptable latency.

In terms of accuracy, the IoU remains above 70% in most cases and degrades modestly under adverse conditions (e.g., a 10–15% drop in IoU between sunny and rainy settings). Precision consistently stays above 78%, and recall stays above 72%, indicating that the model remains effective in identifying key structures such as towers and transmission lines.

These results confirm that the proposed method achieves strong robustness to environmental variability and delivers real-time performance, making it well suited for large-scale power grid inspection tasks in dynamic and unpredictable outdoor conditions.

## 5. Conclusions

This paper proposes a semantic segmentation method for power grid tower point clouds based on multi-sensor fusion and deep learning. Experimental validation demonstrates that the method achieved an overall accuracy of 0.984 in the binary segmentation task. Environmental background recognition achieved the highest precision with an IoU of 0.983, while power tower structures achieved an IoU of 0.731. In the more challenging three-class segmentation task, the model maintained a high accuracy of 0.979. Particularly, in recognizing elongated structures, such as transmission lines, the high recall rate of 0.959 demonstrates the model’s reliable detection capability. These results validate the effectiveness of the proposed method in practical application scenarios.

In-depth analysis reveals that the method exhibits good adaptability in processing targets of different scales, maintaining both the integrity of large-scale structures and the accuracy of detailed features. However, some limitations remain in this study, including factors such as limited training data, scene scale, uneven point cloud distribution, and class imbalance. These issues also indicate directions for future research.

To address these limitations in future work, several promising strategies can be explored. For the limited training data challenge, data augmentation techniques such as random rotation, scaling, jittering, and point cloud mixing could effectively expand the dataset diversity without requiring extensive new data collection. Transfer learning from models that are pre-trained on larger generic point cloud datasets could also mitigate data scarcity issues by leveraging knowledge from related domains. To tackle uneven point cloud distribution, adaptive sampling strategies that assign higher importance to sparse regions (particularly transmission lines and tower extremities) during training could improve feature extraction in underrepresented areas. A multi-resolution approach that processes the point cloud at different scales could also help maintain context while capturing fine details in sparse regions. For class imbalance problems, which are intrinsic to power grid scenes where background points (85%) significantly outnumber tower (10%) and line (5%) points, advanced loss functions like focal loss or class-weighted cross-entropy could be implemented to place greater emphasis on minority classes during training. Additionally, targeted oversampling or synthetic point generation for underrepresented classes could help balance the distribution. These methodological improvements, combined with the optimization of network architectures specifically for linear structures like power lines, represent promising directions for enhancing the performance and robustness of point cloud segmentation in power grid scenarios.

This research presents a comprehensive solution to power grid tower point cloud segmentation through the integration of multi-sensor fusion and deep learning techniques. The approach demonstrates significant potential for practical deployment in intelligent power grid inspection and monitoring applications. By combining established methodologies in a novel system-level framework specifically optimized for power infrastructure, this work advances the field of large-scale point cloud segmentation for critical infrastructure applications.

## Figures and Tables

**Table 1:** Computational efficiency comparison of different sampling methods (10^6^ points).

Sampling Method	Sampling Time (s)	Memory Usage (GB)
FPS	>200	3.2
DS	~10	1.8
RS	0.004	0.5

**Table 2:** Dataset characteristics and model parameters.

Parameter Category	Description
Dataset Characteristics	Number of scenes: 6
	Total points per scene: ~10^7^
	Class distribution:
	- Background: 85%
	- Tower structure: 10%
	- Transmission lines: 5%
	Environmental conditions: flat terrain, mountainous areas, sunny and cloudy
Data Acquisition Parameters	LiDAR: LIVOX AVIA
	- FOV: 70.4° × 77.2°
	- Range: 450 m
	Camera: D345i binocular depth camera
	- Resolution: 1920 × 1080
	Point density: ~1000–2000 points/m^2^
	(near tower structures)
FAST-LIO Parameters	Mapping frequency: 10 Hz
	Voxel size: 5 cm
	IMU integration rate: 200 Hz
	Feature extraction: ~100 features/scan
RandLA-Net Parameters	Random sampling ratio: 0.25 (per layer)
	Number of neighbors (K): 16
	Feature dimensions: [16, 64, 128, 256]
	Attention MLP dimension: 8
	Batch size: 4
	Learning rate: 0.01
	Optimizer: Adam
	Training epochs: 50 (with early stopping)
	Loss function: cross-entropy

**Table 3:** Binary segmentation performance evaluation results.

	IOU	Precision	Recall	F1 Score
Background	0.983281	0.995242	0.987925	0.991570
Tower	0.730715	0.790575	0.906109	0.844408
Miou	0.571332			
Accuracy	0.984006			

**Table 4:** Three-class segmentation performance evaluation results.

	IOU	Precision	Recall	F1 Score
Background	0.981580	0.992479	0.988935	0.990704
Tower	0.671363	0.908176	0.720254	0.803372
Lines	0.514605	0.526301	0.958605	0.679524
Miou	0.541887			
Accuracy	0.979172			

**Table 5:** Computational efficiency comparison of different methods for processing large-scale power tower point clouds.

Method	Processing Time (s)	Parameters (M)	Maximum Points per Pass (M)
PointNet	192	0.8	0.49
PointNet++	289	0.97	0.58
SPG	435	0.25	0.45
RandLA-Net (Ours)	185	1.24	1.03

**Table 6:** Segmentation accuracy comparison of power tower point clouds.

Method	Binary mIoU (%)	Three-Class mIoU (%)
PointNet	61.6	53.6
PointNet++	66.0	62.1
SPG	58.9	52.4
RandLA-Net (Ours)	73.1	67.1

**Table 7:** Segmentation performance and runtime under different environmental scenarios and weather conditions.

Scenario	Weather	Points per Second	Runtime (s)	IoU (%)	Precision (%)	Recall (%)
Urban	Sunny	1.3M	6.3	80.2	90.7	88.3
Urban	Cloudy	1.3M	6.7	78.4	89.3	87.8
Urban	Rainy	1.3M	7.5	72.7	84.6	80.6
Mountain	Sunny	1.5M	5.3	75.9	87.3	85.3
Mountain	Cloudy	1.5M	5.2	70.3	85.6	83.5
Mountain	Rainy	1.5M	6.6	67.5	80.4	78.7
Forest	Sunny	1.1M	7.3	70.3	84.5	80.9
Forest	Cloudy	1.1M	8.5	68.1	83.8	78.1
Forest	Rainy	1.1M	9.6	64.3	78.1	72.2

## Data Availability

The data presented in this study are available on request from the corresponding author.
